# Ruptured metastatic liver tumor secondary to a thymoma: a case report

**DOI:** 10.1093/jscr/rjab341

**Published:** 2021-08-14

**Authors:** Takeshi Utsunomiya, Katsunori Sakamoto, Daiki Tsukamoto, Mikiya Shine, Yusuke Nishi, Takashi Matsui, Kei Tamura, Hitoshi Inoue, Akihiro Takai, Kohei Ogawa, Kotaro Sunago, Yoichi Hiasa, Nobuhiko Sakao, Hisayuki Shigematsu, Yoshifumi Sano, Mie Kurata, Mana Fukushima, Riko Kitazawa, Sohei Kitazawa, Yasutsugu Takada

**Affiliations:** Department of Hepato-Biliary-Pancreatic and Breast Surgery, Ehime University Graduate School of Medicine, Toon, Ehime, Japan; Department of Hepato-Biliary-Pancreatic and Breast Surgery, Ehime University Graduate School of Medicine, Toon, Ehime, Japan; Department of Hepato-Biliary-Pancreatic and Breast Surgery, Ehime University Graduate School of Medicine, Toon, Ehime, Japan; Department of Hepato-Biliary-Pancreatic and Breast Surgery, Ehime University Graduate School of Medicine, Toon, Ehime, Japan; Department of Hepato-Biliary-Pancreatic and Breast Surgery, Ehime University Graduate School of Medicine, Toon, Ehime, Japan; Department of Hepato-Biliary-Pancreatic and Breast Surgery, Ehime University Graduate School of Medicine, Toon, Ehime, Japan; Department of Hepato-Biliary-Pancreatic and Breast Surgery, Ehime University Graduate School of Medicine, Toon, Ehime, Japan; Department of Hepato-Biliary-Pancreatic and Breast Surgery, Ehime University Graduate School of Medicine, Toon, Ehime, Japan; Department of Hepato-Biliary-Pancreatic and Breast Surgery, Ehime University Graduate School of Medicine, Toon, Ehime, Japan; Department of Hepato-Biliary-Pancreatic and Breast Surgery, Ehime University Graduate School of Medicine, Toon, Ehime, Japan; Department of Gastroenterology and Metabology, Ehime University Graduate School of Medicine, Toon, Ehime, Japan; Department of Gastroenterology and Metabology, Ehime University Graduate School of Medicine, Toon, Ehime, Japan; Departments of Thoracic Surgery, Ehime University Graduate School of Medicine, Toon, Ehime, Japan; Departments of Thoracic Surgery, Ehime University Graduate School of Medicine, Toon, Ehime, Japan; Departments of Thoracic Surgery, Ehime University Graduate School of Medicine, Toon, Ehime, Japan; Department of Pathology, Ehime University Proteo-Science Center and Graduate School of Medicine, Toon, Ehime, Japan; Division of Diagnostic Pathology, Ehime University Hospital, Toon, Ehime, Japan; Division of Diagnostic Pathology, Ehime University Hospital, Toon, Ehime, Japan; Department of Molecular Pathology, Ehime University Graduate School of Medicine, Toon, Ehime, Japan; Department of Hepato-Biliary-Pancreatic and Breast Surgery, Ehime University Graduate School of Medicine, Toon, Ehime, Japan

## Abstract

We report a case of rupture of a synchronous metastatic liver tumor secondary to a thymoma. A 56-year-old woman was referred to our hospital with acute abdomen. Computed tomography (CT) revealed a 10 cm diameter tumor in the left lateral segment of the liver, together with ascites, which was suggestive of intra-abdominal bleeding. She was in stable condition and hemostasis was confirmed by angiography. CT also revealed a mass in the anterior mediastinum. Elective laparoscopic left lateral segmentectomy was performed to make a pathological diagnosis and for radical resection. No peritoneal dissemination was observed and the liver tumor was curatively resected. The patient subsequently underwent thymectomy. The pathological diagnoses were thymoma with the liver metastasis. Currently, at 30 months post-treatment, she has had no tumor recurrence. Rupture of a metastatic liver tumor secondary to a thymoma is a rare condition; careful preoperative management and aggressive treatment might improve the patient’s prognosis.

## INTRODUCTION

Tumor metastasis from a thymoma to the liver is relatively rare [1], and rupture of such a tumor is even more rare, with only one such case being previously reported [2]. The rupture of the tumor is a more likely situation in patients with hepatocellular carcinoma or lymphoma [3–6]. In addition, preoperative radiological diagnosis of a metastatic liver tumor secondary to a thymoma is difficult because no specific findings have been previously reported [1]. On the other hand, treatment of metastatic thymoma using a multidisciplinary approach, including radical resection, might result in favorable long-term outcomes in some patients [7]. We report a case of rupture of a synchronous metastatic liver tumor from a thymoma.

## CASE REPORT

**Figure 1:**
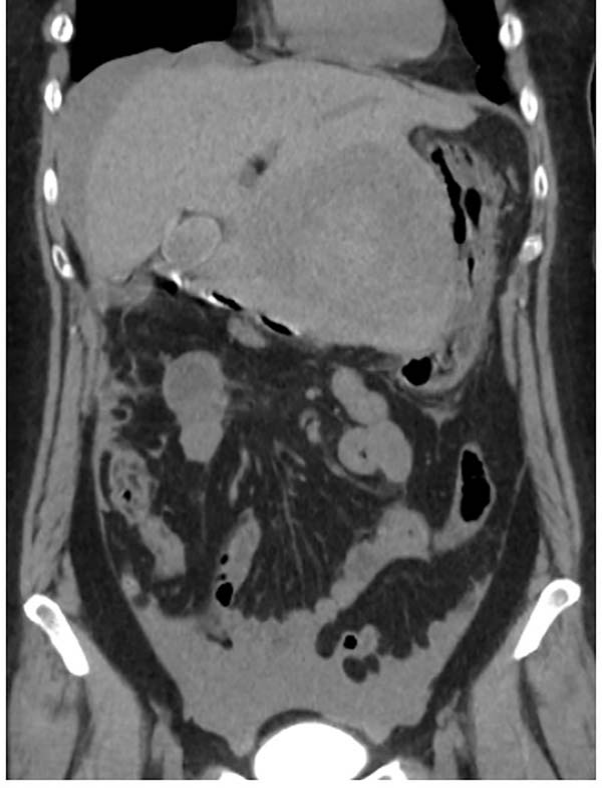
Abdominal CT at the onset of symptoms suggestive of acute abdomen. A 10 cm diameter liver tumor was seen to protrude from the left lateral segment of the liver and high-density ascites was identified around the liver.

**Figure 2:**
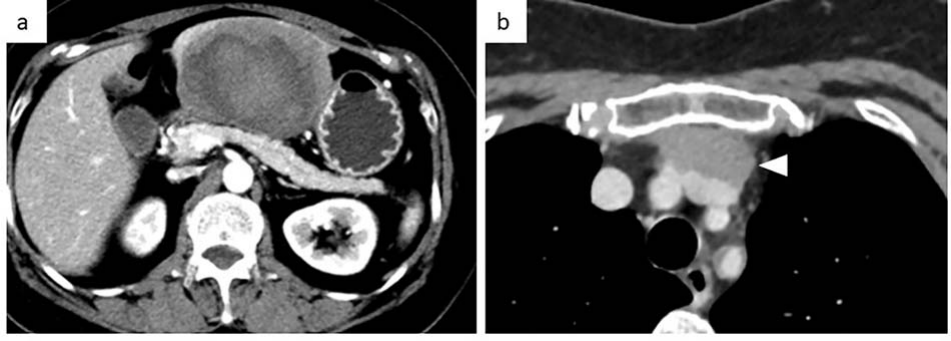
Contrast-enhanced CT. (**a**) Abdominal CT: a solitary liver tumor, 10 cm in diameter, was located in the left lateral segment of the liver. The tumor had a clear margin, with tumor enhancement in both the arterial and portal phases. (**b**) Chest CT: an enhanced tumor with an irregular margin was detected in the anterior mediastinum. The tumor had a maximum diameter of 4.5 cm.

**Figure 3:**
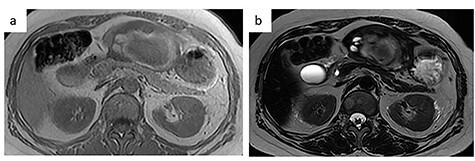
MRI. (**a**) T1-weighted image: low-intensity area suggestive of intratumor hemorrhage. (**b**) T2-weighted image: the tumor had heterogenous intensity.

**Figure 4:**
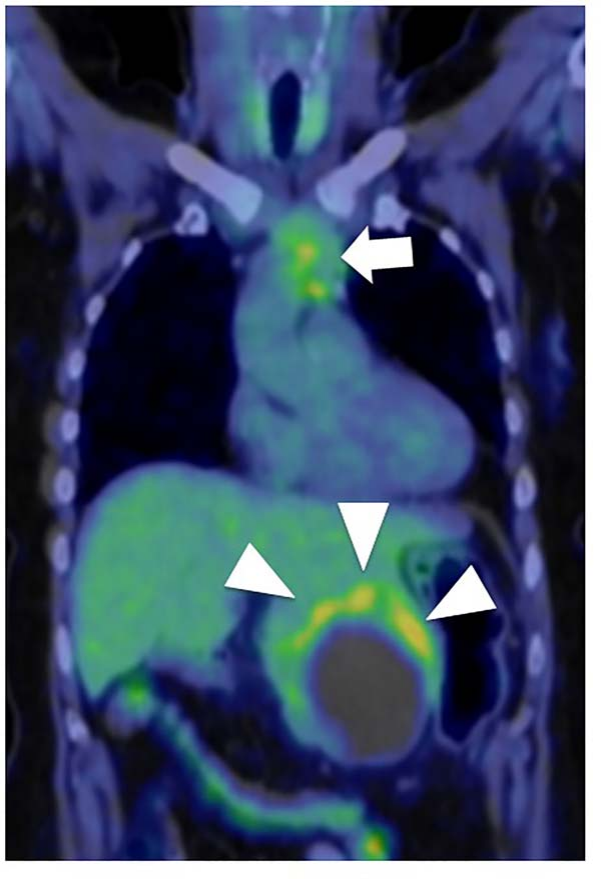
Fluorodeoxyglucose positron emission tomography. Maximum standard uptake value of the liver tumor (arrow head) was 4.8 and that of the anterior mediastinal tumor (arrow) was 3.9. Neither lymph node metastasis nor other distant metastasis was detected.

**Figure 5:**
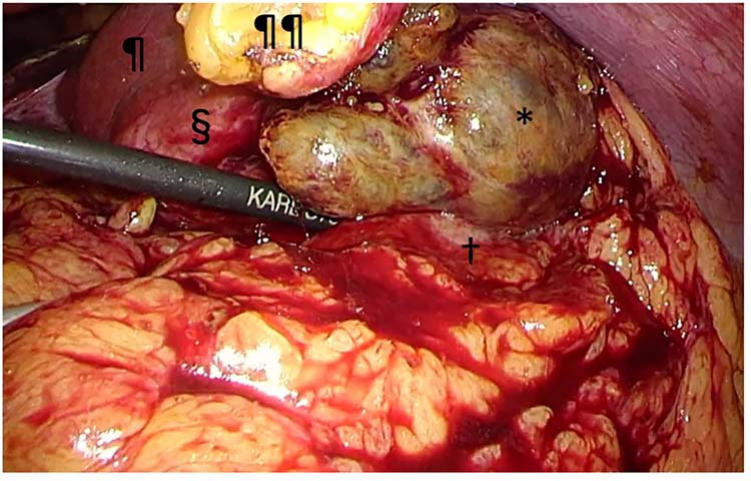
Intraoperative findings. The tumor (§) with its protruding content (*) was adherent to the greater omentum and stomach (†). The tumor was bluntly dissected and no peritoneal dissemination was observed. ^¶^liver; ^¶¶^divided round ligament.

**Figure 6:**
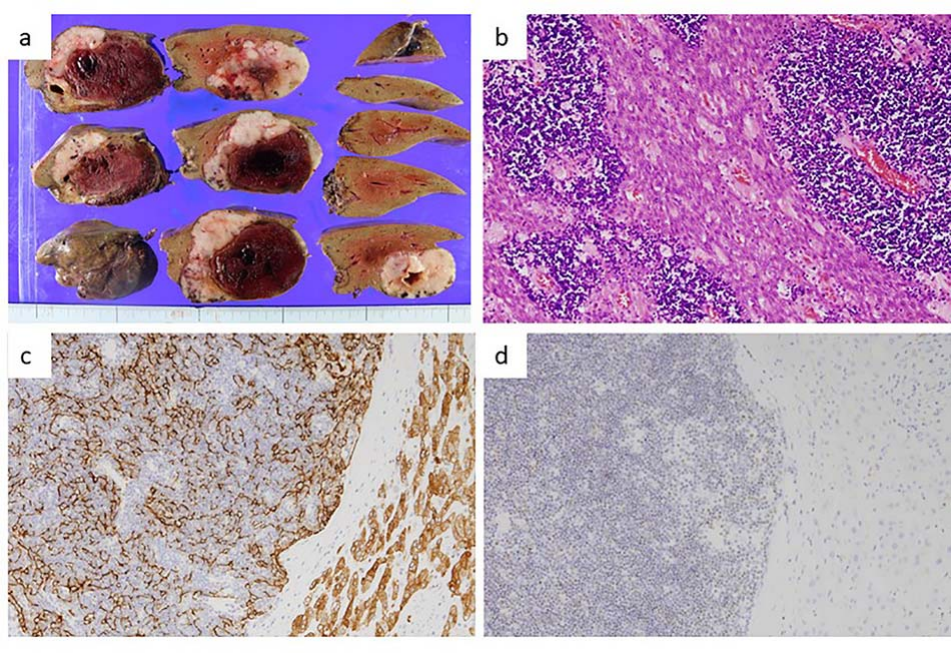
Pathological findings of the liver tumor. (**a**) Gross view of a cross-section of the liver tumor. The lesion, measuring 7.5 × 7.5 × 4 cm, was seen to consist of a grayish-white solid tumor with a massive intratumor hematoma. (**b**) Hematoxylin–eosin staining of the liver tumor (×200) showed a lobulated architecture with intersecting bands and dense infiltration of small lymphocytes. Eosinophilic bands containing epithelial cells with wide cytoplasm and an oval nucleus, resembling medullary cells of the thymus, were visible. These findings were compatible with metastasis of a type AB thymoma. (**c**) Immunohistochemical staining for cytokeratin (AE1/AE3) (×400). Epithelial cells positive for cytokeratin (AE1/AE3) were arranged in a fine meshwork. (**d**) Immunohistochemical staining for terminal deoxynucleotidyl transferase (TdT) (×200). The infiltrating lymphocytes were positive for TdT, which is a marker of immature T-cells.

A 56-year-old woman presented to another hospital with acute abdomen. Computed tomography (CT) examination demonstrated a tumor 10 cm in diameter that protruded from the left lateral segment of the liver, with evidence suggestive of rupture of the liver tumor (Fig. 1). Since the patient was in good general condition, she was referred to our hospital for further evaluation. Her blood biochemical parameters when she was referred to our hospital were hemoglobin: 11.6 g/dl, albumin: 3.4 g/dl, γ-globulin: 18.5% and anti-acetylcholine receptor antibody: <0.3 nmol/L. Dynamic CT presented a liver tumor 10 cm in diameter, which was located at the left lateral segment of the liver, with a clear margin. A mass with an irregular margin was also identified in the anterior mediastinum (Fig. 2). On percutaneous angiography, since obvious extravasation of the contrast medium could not be identified, the arteries of the left lateral segment were embolized to prevent re-bleeding. Although a definitive preoperative diagnosis could not be made using magnetic resonance imaging (MRI) (Fig. 3), fluorodeoxyglucose positron emission tomography (FDG) showed increased FDG uptake in both the hepatic and anterior mediastinal tumors (Fig. 4). Although the liver tumor was suspected to be metastasis secondary to a thymoma based on the clinical findings, percutaneous tumor biopsy was performed to confirm the pathological diagnosis. The biopsy suggested malignant T-cell lymphoma or metastatic thymoma. In order to prevent re-rupture of the hepatic tumor, to confirm the pathological diagnosis and to potentially achieve a curative resection, the patient underwent laparoscopic left lateral segmentectomy (Fig. 5). Although the tumor was found to be adherent to the stomach, blunt dissection was possible. In addition, no peritoneal dissemination was detected. The surgical duration was 212 min and estimated blood loss was 50 ml. The liver tumor was pathologically diagnosed as metastatic thymoma type AB (Fig. 6). The patient’s postoperative course was uneventful and she subsequently underwent radical thymectomy 3 months after the liver resection. The thymic tumor was pathologically diagnosed as thymoma type B2. Currently, 30 months after thymectomy, she remains free from tumor recurrence.

## DISCUSSION

Metastatic lesions from thymomas are mainly due to intrathoracic dissemination, and their hepatic metastasis is rare [1]. Khandelwal *et al*. [1] reported that liver metastasis of thymoma occurred in about 4.8% of 62 thymoma cases. Rupture of a metastatic liver tumor, as seen in our case, is even rarer. To the best of our knowledge, only one previous report of rupture of a metastatic liver tumor from thymoma has been reported [2]. In that case, the liver metastasis was detected metachronously from the primary thymic tumor and liver resection was performed after four cycles of chemotherapy [2]. In addition, in that case, no peritoneal dissemination was detected, similar to our case. They performed preoperative chemotherapy for the liver metastasis of thymoma because of metachronous presentation of the disease, whereas we performed radical surgery to prevent re-rupture of the hepatic lesion and to confirm the pathological diagnosis, with subsequent thymectomy without preoperative chemotherapy for the synchronously presenting tumors. Similar to hepatocellular carcinoma [8], tumor rupture of liver metastases from thymoma might not always result in peritoneal dissemination. Hence, radical resection should be considered even for patients with tumor rupture.

Making a preoperative diagnosis was difficult in the present case since typical imaging findings have not been previously reported and tumor rupture is very rare. Although we did not hesitate to perform tumor biopsy in the present case because the tumor had already ruptured, performing tumor biopsy should be carefully considered because of the risk of peritoneal dissemination [9]. In addition, tumor biopsy might confuse the preoperative diagnosis, as in our case in which the pathological pattern was different between the primary site and site of metastasis. In this patient, we considered T-cell lymphoma as one of the differential diagnosis based on the results of pathological evaluation of the biopsy specimen, since rupture of a malignant lymphoma is more likely than that of a thymoma [4–6]. However, we suspected synchronous liver metastasis from a thymoma based on the overall clinical features.

In the present case, contrast-enhanced CT demonstrated two-layered enhancement of the liver tumor. Considering the gross findings of the liver tumor, the inner layer consisted of a hematoma, and the outer layer was formed by compression of the tumor into a crescent shape by the intratumor hematoma, as seen on MRI. Since thymomas, including those that metastasize to the liver are soft, they can rupture easily and can be easily compressed by intratumor hematoma.

The 5-year survival rate in patients with thymoma was previously reported as 70.6% for Stage IVA and 52.8% for Stage IVB tumors [3]. Radical resection is essential to improve the long-term prognosis of cases with advanced thymoma [10]. In a previous study, the radical resection rate of Masaoka Stage IVB thymomas was 47%, and the recurrence rate was 44% in all cases that underwent adjuvant therapy [11]. There are some phase II trials and retrospective reports about adjuvant therapy for post-resection thymomas. However, randomized controlled studies describing adjuvant therapy have not been reported. Hence, adjuvant therapy for thymomas is not standardized in Japan and we did not administer adjuvant therapy after the radical resection in our case. The patient has been closely followed-up till now, with currently no obvious intrahepatic recurrences or peritoneal dissemination 30 months after the treatment.

In conclusion, careful preoperative management and aggressive treatment might improve patient prognosis in cases with metastatic liver tumors secondary to thymomas.

## CONFLICT OF INTEREST STATEMENT

None declared.
